# Comparison of Intra-articular Haematoma Block and Procedural Sedation for the Manipulation of Closed Ankle Fracture Dislocations: A Cross-Sectional Study

**DOI:** 10.7759/cureus.80003

**Published:** 2025-03-03

**Authors:** Mahmoud Elmesalmi, Zeid Morcos, John Mcfall, Fadi Hindi, Khaled F Al-Kharouf, Togay Koç

**Affiliations:** 1 Trauma and Orthopaedics, St George's University Hospitals NHS Foundation Trust, London, GBR; 2 Trauma and Orthopaedics, Queen Alexandra Hospital, Portsmouth, GBR; 3 Trauma and Orthopaedics, University Hospital Southampton, Southamton, GBR

**Keywords:** ankle fracture dislocation, cost-effective therapy, intra-articular hematoma block, mua, procedural sedation

## Abstract

Background

Ankle fracture dislocations are commonly reduced in the emergency setting under procedural sedation (PS), which requires trained clinicians and monitoring. This study aimed to evaluate the patient-reported efficacy of intra-articular haematoma block (IAHB) as an analgesic alternative to PS for the closed reduction of ankle fracture dislocations.

Methods

Data from patients with displaced ankle fractures requiring manipulation between October 2020 and April 2021 were analysed. Patients who received IAHB were compared to those who received PS. IAHB involved the injection of 10 mL of 1% lignocaine into the joint space.

Results

Twenty-eight patients received PS, and 25 received IAHB. There were no statistically significant differences in visual analogue scale (VAS) scores before, during, or after treatment (p > 0.05). First-attempt reductions were successful in 76% of IAHB patients compared to 82.1% of PS patients. IAHB was associated with lower medication costs and a shorter time to manipulation.

Conclusion

IAHB is a cost-effective and safe alternative to PS for managing ankle fracture dislocations.

## Introduction

Orthopaedic injuries requiring manipulation can be resource-intensive for emergency departments (EDs), especially when conscious procedural sedation (PS) is needed [1]. The Royal College of Emergency Medicine [2] mandates that conscious sedation be administered by an appropriately trained clinician with a skilled assistant, along with safe monitoring. Thus, the availability of appropriately trained clinicians and a monitored bed space in our increasingly busy EDs can impact manipulation times. Patient factors, such as fasting status and comorbidities, can also affect the suitability of PS.

With the devolution of musculoskeletal practice in our EDs over the years, advanced and emergency nurse practitioners (ANPs/ENPs) [3] now possess the skills to perform manipulations, but the bottleneck remains the anaesthesia. Haematoma blocks for distal radius fractures are routinely performed nationwide with good results [4,5].

In 2002, Miller et al. prospectively randomized 30 patients with isolated glenohumeral joint dislocations to undergo closed reduction with the use of either intra-articular lidocaine or intravenous sedation [6]. There were no significant differences in pain scores (p = 0.37) or the ability to reduce the shoulders. However, patients receiving PS spent significantly longer in the ED (185 vs 75 minutes), with an average cost of $97.64 compared to $0.52 for the lidocaine group [6].

White et al. conducted what is believed to be the first prospective randomized trial comparing intra-articular haematoma blocks (IAHBs) with conscious sedation for 42 closed reductions of ankle fracture dislocations [4]. Using a visual analogue scale (VAS) score, both groups achieved a statistically significant reduction in pain (IAHB, 9.2 to 3.6; PS, 9.3 to 4.1) with no significant difference between the two methods. They also stated that both methods provided similar levels of analgesia during the manipulation, but offered no supporting data. They did not find a significant difference in the time taken to deliver patient care. The IAHB group required six further manipulations, while the PS group required two (p = 0.15). White et al. demonstrated that intra-articular injection of local anaesthetics delivers a similar degree of analgesia to conscious sedation [4].

Furia et al. and Alioto et al. together report using IAHB on 23 patients without any episodes of associated infection [5,7]. Additionally, local anaesthetic chondrotoxicity has been well documented in vitro, with local anaesthetics exhibiting dose- and time-dependent cytotoxic effects [5,7].

The primary outcome of this study was to assess the patient-reported efficacy of IAHB as analgesia for the closed reduction of ankle fracture dislocations in a large, busy British NHS Hospital, compared to PS. Secondary outcomes included time to manipulation, efficiency of patient flow through the ED, economic evaluation, and manipulation success.

## Materials and methods

This study included a total of 53 patients presenting with displaced ankle fractures requiring manipulation under anaesthesia (MUA). A cross-sectional design was employed, with prospective data collection for 25 patients undergoing IAHB from October 2020 to April 2021 and a retrospective analysis of 28 patients who underwent PS for ankle fracture manipulation over a six-month period from March to August 2020. All patients underwent a comprehensive medical history assessment, including injury mechanism and cause, and a clinical examination focusing on tenderness, swelling, and ankle joint movement limitations. Anteroposterior (AP) and lateral X-rays were performed prior to closed reduction. Pain was evaluated using VAS [8] before, during, and after reduction. Data included the number of clinicians involved, time from initial X-ray to reduction (or from the presentation if initially seen at a satellite minor injury unit (MIU)), success rate of reduction, and number of reduction attempts. Medications and their respective volumes were documented to calculate the total cost of medications. The cost was determined by multiplying the unit cost of each medication (per mg, mL, or mcg) by the quantity used. The following unit costs were applied: ketamine at £0.014 per mg, propofol at £0.015 per mg, fentanyl at £0.0143 per mcg, and lignocaine at £0.56 per 10 mL.

Data for the IAHB group were collected from written forms filled out by the clinician, while data for the PS group were collected from electronic ED documentation. The X-rays were compared using Picture Archiving and Communication System (PACS).

The IAHB technique involved injecting 10 mL of 1% lignocaine into the joint space via a medial approach adjacent to the tibialis anterior tendon, performed by a trauma and orthopaedic middle-grade clinician or ANP trained in the procedure, while PS was conducted in the ED by emergency physicians using agents such as propofol, ketamine, or benzodiazepines based on the clinical assessment. The study population included adult patients aged 18 years and older, with displaced ankle fractures requiring MUA, excluding those with open fractures, skin-threatening injuries, neurovascular compromise, or polytrauma. 

Statistical analysis was conducted using IIBM SPSS Statistics for Windows, Version 27 (Released 2020; IBM Corp., Armonk, NY, United States). Categorical variables were presented as counts and percentages and compared between groups using Pearson’s chi-square test or Fisher’s exact test when more than 25% of cells had a count of <5. Quantitative variables, such as pain scores, were analysed using the Wilcoxon signed-rank test, while temporal variables were assessed using the Mann-Whitney U test. A p-value of <0.05 was considered statistically significant. 

This study employs a combination of retrospective and prospective audit methodologies using non-identifiable data, ensuring no risk to the patients involved. Consequently, ethical approval was not required. The study was conducted in the Orthopaedics Department at Queen Alexandra Hospital, Portsmouth, Hampshire, UK, and was registered with the institutional governance team as a service evaluation.

## Results

Patient characteristics, clinician involvement, and time metrics

**Figure 1:**
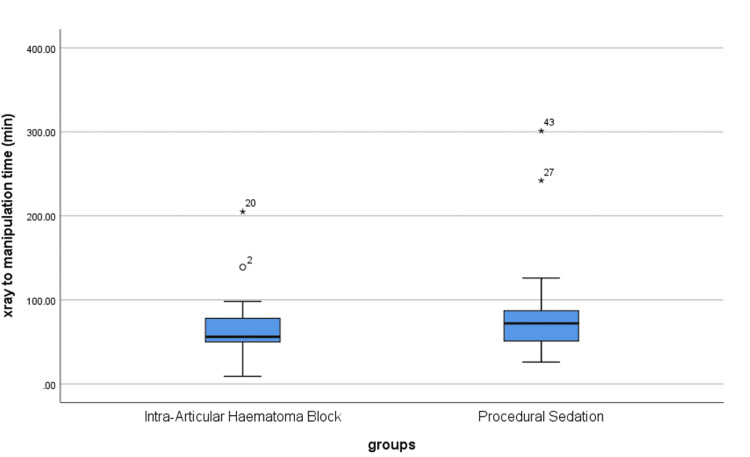
Time from X-ray to manipulation in both groups

**Figure 2:**
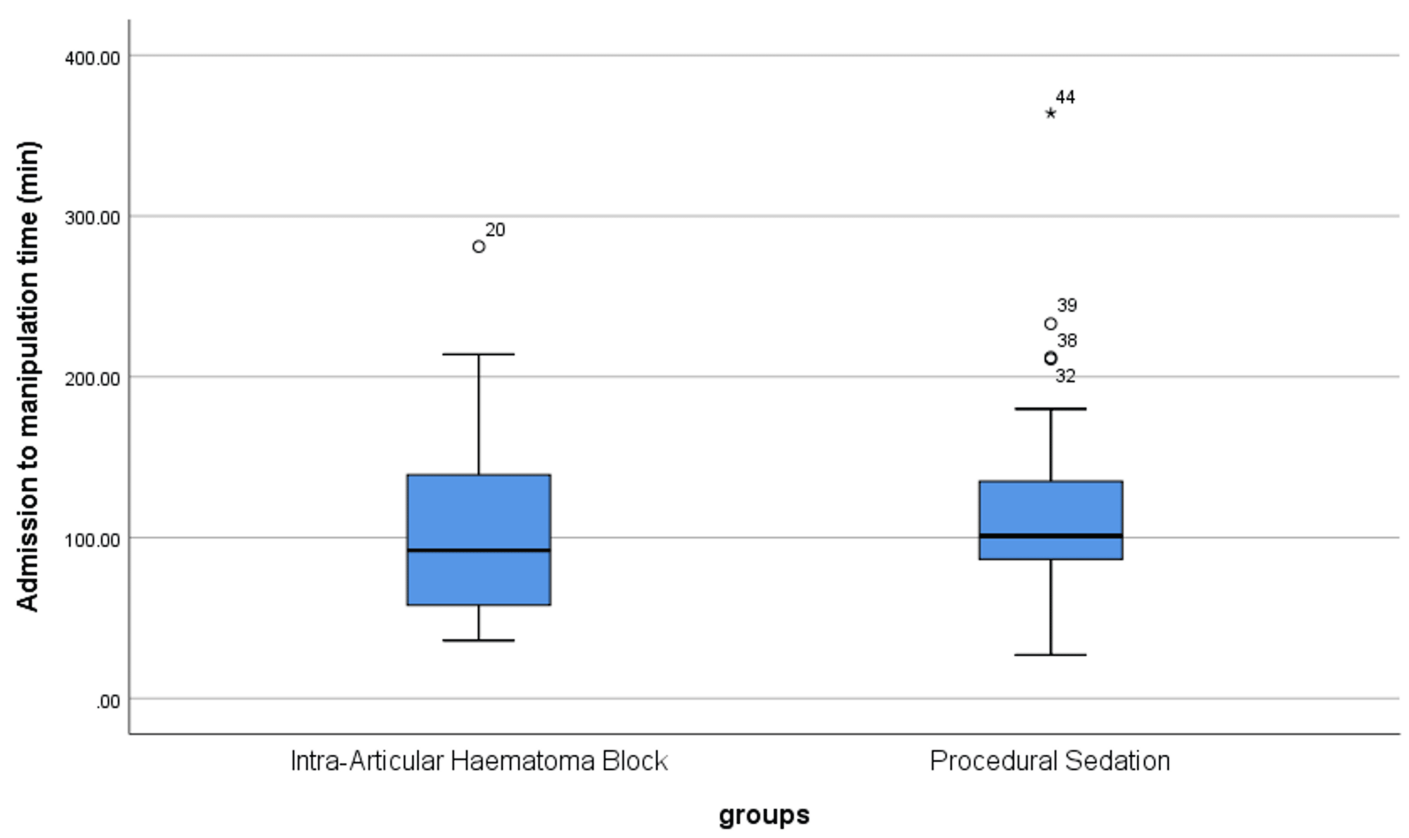
Time from admission to manipulation in both groups

The mean age was 57.8 ± 19.16 years in the IAHB group and 55.14 ± 17.21 years in the PS group (p = 0.59). The number of clinicians involved was significantly higher in the PS group than in the IAHB group (p = 0.002). There were no statistically significant differences between both groups regarding time from X-ray to manipulation, time from admission to manipulation, and time spent in the ED (p > 0.05) (Table 1, Figure 1, Figure 2).

**Table 1:** Patient characteristics, clinician involvement, and time metrics for both groups Continuous data were represented as mean± SD unless mentioned otherwise, Mann-Whitney U test, independent-sample t-test, chi-square, Fisher's exact test.

	Intra-articular haematoma block (N = 25)	Procedural sedation (N = 28)	p-value
Age in years	57.8 ± 19.16	55.14 ± 17.21	0.590
Number of clinicians involved			0.002
Two	1 (4.0%)	0 (0.0%)	
Three	22 (88.0%)	14 (50.0%)	
Four	2 (8.0%)	9 (32.1%)	
Five	0 (0.0%)	5 (17.9%)	
On-call orthopaedic team involved	20 (80.0%)	21 (75.0%)	0.660
Median (IQR) X-ray to manipulation time (minutes)	56 (48-78)	72 (50.5- 87)	0.173
Median (IQR) admission to manipulation time (minutes)	92 (57.5- 139)	101 (86.25- 135)	0.387
Time spent in the ED (minutes)	286.65 ± 126.88	247.89 ± 89.49	0.230

Pain assessment using VAS scores

**Figure 3:**
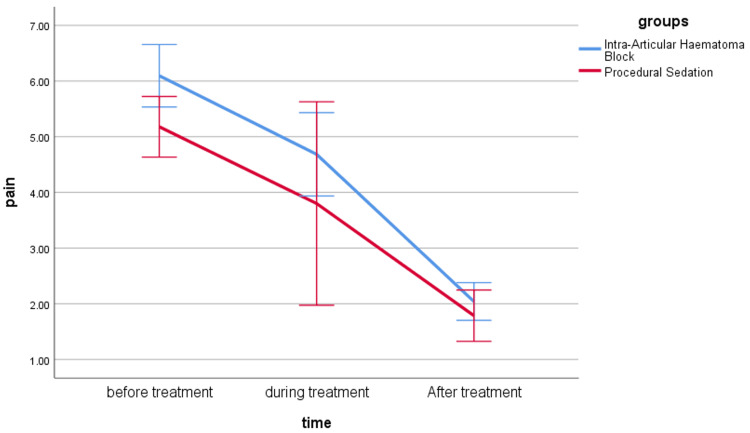
Line chart for repeated measurements for VAS pain score in both groups

There were no statistically significant differences between both groups in VAS scores before, during, and after treatment (p > 0.05) (Table 2, Figure 3).

**Table 2:** Assessment of pain using VAS score in both groups Data were represented as mean ± SD; mixed linear model adjusted with the Bonferroni test.

	Intra-articular haematoma block	Procedural sedation	p-value
Preoperative VAS pain	6.10 ± 2.80	5.18 ± 2.88	0.250
Pain during reduction	4.68 ± 3.74	3.80 ± 4.09	0.640
Postoperative VAS pain	2.04 ± 1.70	1.79 ± 2.44	0.670

Reduction success and further manipulations

There was no statistically significant difference in satisfactory first-attempt reduction rate between the IAHB group (19 patients, 76.0%) and PS group (23 patients, 82.1%) (p = 0.582). In the IAHB group, six patients had unsatisfactory first attempts at reduction. Five of the six went on to have further manipulation under the same block. Three of them were satisfactory at the second attempt, while one underwent MUA and plaster the following day and the other failed manipulation under PS and went for fixation the following morning. In the PS group, five patients had unsatisfactory first attempts at reduction. One had two further episodes of PS, but the reduction was still unsatisfactory, so the patient underwent fixation the same day. The remaining four patients had no more attempts at reduction under PS and all went on to have external fixation (three patients) or open reduction and internal fixation (one patient) within 24 hours. There was no significant difference in the number of unsatisfactory reductions between the groups (Table 3).

**Table 3:** Satisfactory reduction in both groups Crosstabs and chi-square test or Fisher's exact test. Data were represented as events (%).

	Intra-articular haematoma block (N = 25)	Procedural sedation (N = 28)	p-value
Satisfactory reduction first attempt			
Yes	19 (76.0%)	23 (82.1%)	0.582
No	6 (24.0%)	5 (17.9%)	
Further manipulation	5 (20%)	1 (3.6%)	0.062
Satisfactory reduction second attempt	3 (12%)	0 (0.0%)	0.061

Medication volume and cost

**Figure 4:**
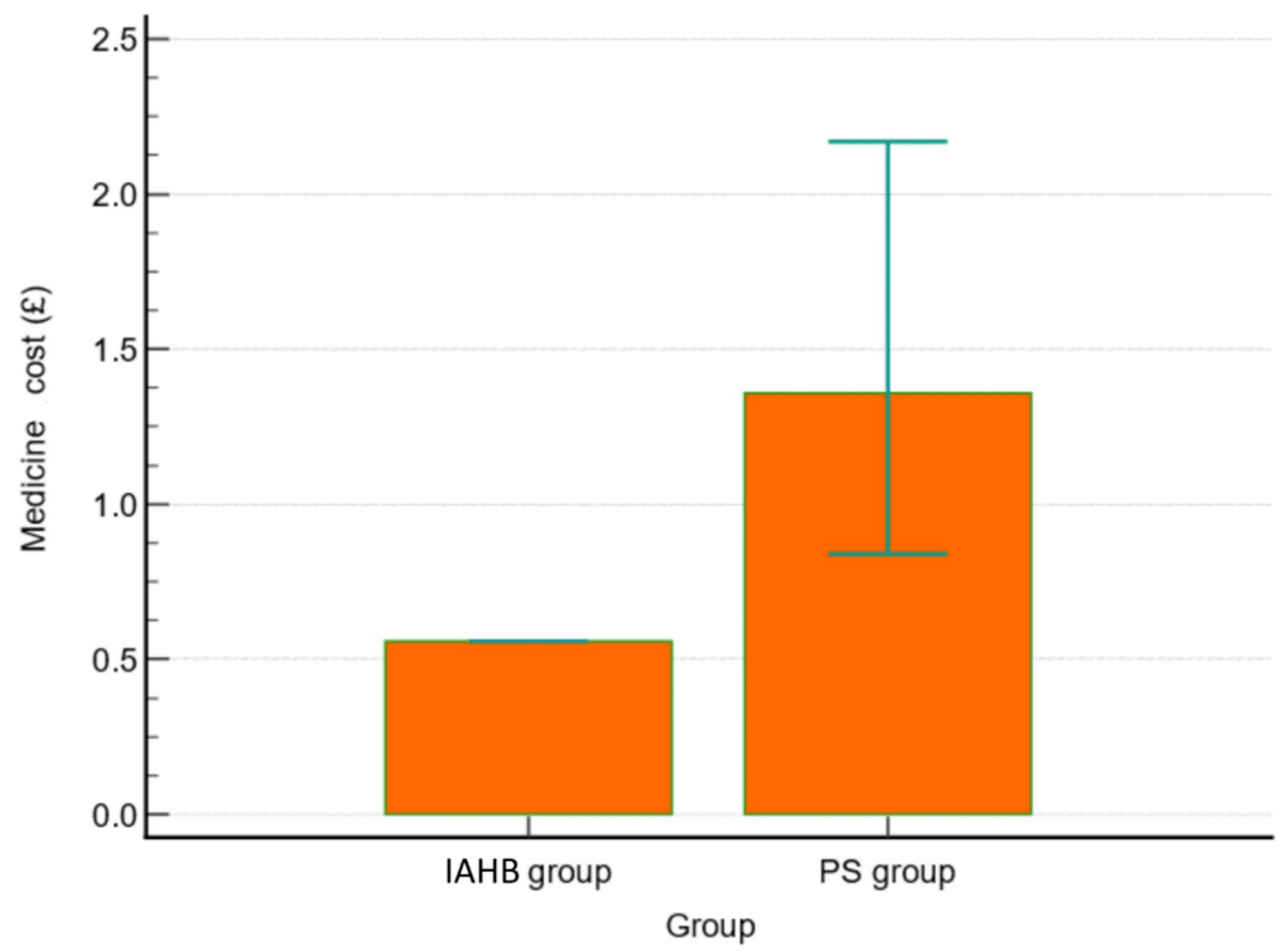
Box plot for total cost of medicines (£) in both groups IAHB: intra-articular haematoma block, PS: procedural sedation.

In the IAHB group, the mean volume of 1% lignocaine used prior to manipulations was 10.2 ± 1.0 ml. In the PS group, 72.7 ± 20.4 mg of ketamine was used in 13 patients, and 79.2 ± 48.6 mcg of fentanyl was used in 13 patients. Additionally, propofol (77.9 ± 47.9 mg) was used in 14 patients. The cost of medications was significantly higher in the PS group, with a median of £1.4 (0.6-4.7), compared to £0.56 (0.56-0.84) in the IHAB group (p < 0.001) (Table 4, Figure 4).

**Table 4:** Medications used and their total cost in both groups Mean ± SD, median (min-max), Mann-Whitney U test.

	Intra-articular haematoma block	Procedural sedation	p-value
1% lignocaine (mL) (n = 25)	10.2 ± 1.0	--	--
Ketamine (mg) (N = 13)	--	72.7 ± 20.4	--
Propofol (mg) (N = 14)	--	77.9 ± 47.9	--
Fentanyl (mcg) (N = 13)	--	79.2 ± 48.6	--
Ketamine (£) (N = 13)	--	1.08 ± 0.53	--
Propofol (£) (N = 14)	--	1.23 ± 0.76	--
Fentanyl (£) (N = 13)	--	1.03 ± 0.8	--
Total cost of medicines (£)	0.56 (0.56-0.84)	1.4 (0.6-4.7)	<0.001

## Discussion

This study found that IAHB provides comparable analgesia to PS for the closed reduction of ankle fracture dislocations. There were no statistically significant differences before, during, and after procedural pain scores between the study groups, aligning with findings from Alioto et al. and White et al. [4,7]. The first-attempt reduction success rate was also similar, with 76% for IAHB and 82.1% for PS. While six patients had a failed first attempt, three of them had satisfactory second-attempt reduction under the same block, while one patient in the IAHB group had an unsatisfactory second attempt under the same block. Notably, another patient in the IAHB group who also had an unsatisfactory second attempt failed even after sedation. In contrast, five patients in the PS group required urgent operative intervention. These results suggest that while both methods are comparable for initial reduction attempts, the IAHB group showed fewer subsequent failures requiring emergency surgery. Our study showed no statistically significant differences between the groups regarding time from X-ray to manipulation, time from admission to manipulation, and time spent in the ED.

Ankle fracture dislocations are common injuries managed in the ED [9,10]. PS carries a risky complication profile, particularly when conducted in hospitals with limited staff and resources, deep sedation in elderly patients with significant comorbidities, or during overnight hours [11]. IAHB is a regional anaesthetic technique that can be used among selected patients to minimize the anticipated complications of PS [12]. IAHB provides an equivalent amount of analgesia to conscious sedation among patients with closed ankle fracture dislocation, with lower cardiovascular risk, reduced cost, and timelier intervention [13]. The first study on the IAHB technique was conducted by Alioto et al. in 1995 [7]. They reported that the haematoma block is a safe, effective, and low-cost procedure for patients with closed ankle fractures [7]. Similar findings were reported by White et al. [4], who conducted a prospective randomized study with 42 patients with ankle fracture dislocation randomly assigned to two groups: continuous sedation and intra-articular lidocaine block. They found that there was no statistically significant difference between the study groups in terms of pain scores [4]. Conversely, our findings were inconsistent with MacCormick et al. [14], who retrospectively reviewed 221 patients who underwent IAHB and 114 patients who underwent PS. They found that first-attempt reduction was significantly lower among the IAHB group (54.8%) compared to the PS group (74%). However, there was only one case of respiratory depression in the PS group. The superiority of IAHB was mainly among patients who had ankle fractures with accompanying joint subluxation [14].

We found that the number of clinicians involved was significantly higher in the PS group than in the IAHB group. The cost of medications was 2.5 times higher in the PS group compared to the IAHB group, with median costs of £1.40 and £0.56, respectively. These findings were comparable to those provided by MacCormick et al. [14], who reported a mean cost of $4 for IAHB versus $220 for PS. They also compared outcomes between orthopaedic surgeons and ED clinicians, showing that successful reduction rates at the first attempt were significantly higher among orthopaedists compared to ED clinicians [14]. We believe that IAHB may have a role in the initial management of ankle fractures in well-equipped, high-volume hospitals in central areas and large cities, as well as during pandemics such as COVID-19, where it offers the advantage of avoiding aerosol-generating procedures. Additionally, it may be useful in low-resourced healthcare facilities, including hospitals with a shortage of medical staff. For patients in whom IAHB is unsuccessful, PS can be considered, potentially reducing overall costs. Advantages of this method include limiting the requirement for designated clinical space and the need for qualified clinicians to administer sedation, as well as the ability to re-manipulate under the same block.

The limitations of this study include being a single-institution study and not categorizing patients based on gender and the severity and extent of ankle injury. Additionally, cost analysis did not include monitoring expenses, operating room utilization, and attending physicians’ fees. Future outcomes, such as whether the patient eventually required an operation or not, were not considered. Furthermore, the absence of gender data is a minor limitation. Additionally, the study design presents a limitation, as one group was assessed prospectively while the other was analysed retrospectively. A fully prospective approach would enhance comparability. Future research should consider a strictly prospective design to strengthen these findings.

## Conclusions

IAHB appears to be an efficacious, safe, and inexpensive technique for providing analgesia during the manipulation of closed ankle fracture dislocations in emergency settings. It is a simple and rapid procedure that can be performed by orthopaedic and emergency care practitioners, making it particularly valuable in busy or resource-limited institutions. The choice of method should be tailored to the patient's comorbidities, as well as the availability of staff and resources. PS carries a risky complication profile particularly when conducted in hospitals with limited staff and resources. We recommend conducting a national-level study to further evaluate the role of IAHB as a timely and accessible intervention for ankle fracture dislocations.
